# The Bodies in Charge of Animal Welfare: What They Do and What They Could Do?

**DOI:** 10.3389/fphys.2018.00391

**Published:** 2018-04-17

**Authors:** Giuliano Grignaschi, Veronica Redaelli, Fabio Luzi, Massenzio Fornasier

**Affiliations:** ^1^Animal Care Unit, Istituto di Ricerche Farmacologiche Mario Negri, Milan, Italy; ^2^Dipartimento di Medicina Veterinaria, Università degli Studi di Milano, Milan, Italy; ^3^Società Italiana Veterinari Animali da Laboratorio (SIVAL), Cremona, Italy

**Keywords:** Italy, bodies in charge, animal welfare, laboratiory animals, legislation as topic

## Abstract

The coming into force of the 2010/63/EU (Directive of the European Parliament and of the Council of 22 September 2010)^1^ Standard, regarding the protection of animals used for scientific purposes, has made it mandatory for all establishments breeding, supplying, and using said animals to have an Animal Welfare Body (AWB). The establishment of a body such as the AWB represents a strong innovation compared to previous regulations (Dir. 86/609/CEE). Building from the key concept of the 3 Rs, European Community legislators acknowledged that the effective safeguard of animal welfare depends in large part on the professional skills of personnel in charge of their care and use. The European Community legislators therefore identify a body inside the institution that houses the animals and entrust it with the task to stimulate and support the practical implementation of the 3 Rs, by informing on technical and scientific developments on the application of said principle and the subsequent training and follow-up training of personnel. The functions assigned by the Standard to the AWB therefore focus on technical-scientific support: to supply advice to personnel in charge of animals concerning their welfare, matters relating to their acquisition, housing, care, and use, and to their integration/adoption (rehoming) at the end of their use. This approach is also emphasized by vesting the AWB with the responsibility to define and review internal monitoring and communication procedures pertaining to the welfare of the animals housed in the establishment, and to follow their development and the outcome of research projects concerning the effects produced on the animals used, supplying advice on activities that could result in possible improvements. Aware of the complexity and sensitivity of the role assigned to the AWB, and of the difficulty to put into practice the directions subject matter of the Standard, The European Commission, in the years following the issue of the Directive, appointed groups of experts with the task to formulate guidelines which would be beneficial both to the establishments and to control authorities of the various Member States and guarantee the implementation of effective and to control authorities of the various Member States and guarantee the implementation of effective and harmonized solutions. (National Competent Authorities for the implementation of Directive 2010/63/EU, http://ec.europa.eu/environment/chemicals/lab_animals/pubs_guidance_en.htm)^2^.

## Transposition of european directive

### Tasks of the animal welfare body

Legislative Degree No. 26/2014 (National Competent Authorities for the implementation of Directive 2010/63/EU)^3^,^4^ which transposes Directive 63/2010, goes back to the fundamental elements regarding the AWB, that is the minimum composition and the responsibilities on the appointment of members (Art. 25). If, however, the tasks and the responsibilities (Art. 26) of the Animal Welfare Body (AWB) are examined in detail, comparing them with the ones defined by Directive 2010/63/EU (*Tasks of the Animal Welfare Body*—Art. 27), the willingness of Italian legislators to entrust the Body with additional tasks is evident.

Among them, the most important, is the formulation of a *motivated opinion* on the applications for authorizing research projects, based on the assessment of a series of elements, that range from checking the correct application of the provisions of the decree, to the evaluation of the adequate training of personnel and especially to the technical-scientific relevance and the harm-benefit ratio. We are dealing with a fundamental innovation, since the Directive entrusted these assessments exclusively to the competent authorities, to be carried out while reviewing the authorization application for the Research Project (art. 38 of the Directive).

It is clear that Italian legislators wanted to introduce an internal preliminary regulatory system, aimed at minimizing, as much as possible, the filing of incomplete or nonconforming applications, and slowing down the revision and approval procedure. Since the guidelines for the formulation of a motivated opinion have not been defined, the prevailing (and most logical) trend calls for the AWB to revise the project, using the same technical-scientific evaluation criteria needed for the authorization (Art. 31 sub-paragraph 4 of Legislative Decree 26/2014).

The information necessary for the technical-scientific assessment must be listed and discussed by the Project Manager in the communication text, drawn-up in conformity with the provisions of annex VI of Legislative Decree 26/2014.

The task of the AWB, specifically because it substantially belongs to the organization in charge of its appointment, is therefore to aid the Project Manager by critically revising the various aspects of the project and guarantee that the assessments listed in the authorization application are clear, consistent, and can be shared. Said revision is more effective if it is based on continuous support to the Research Project manager during the preparation stage of the authorization application, which results in the best possible compliance with the concept of the 3 Rs.

The role of the AWB, as the agency in charge of the technical-scientific assessments of first level Projects, followed by a second level assessment by competent Authorities, must be interpreted in such a way. After all, it is hard to imagine the AWB to express, as part of the authorization application, a motivated negative opinion, or an opinion non-conforming to the assessments of the Project Manager.

### Functions of members of the AWB

The European Commission guidelines regarding the functions of the members of the AWB, were also officially implemented by the Member States in February 2014 (National Competent Authorities for the implementation of Directive 2010/63/EU) and, even if not binding, clarify many aspects related to the professional profile of the Supervisor of Animal Welfare and the infrastructures (the former Supervisors of the User Establishment) and the Appointed Veterinarian. For the latter, professional associations got to work to have the meaning of the definition “Veterinarian skilled in laboratory animals” (Poirier et al., 2015), introduced by the provision (Art. 24), clarified.

There are no indications regarding the functions of the scientific member provided only for the user establishments, for which there is a generic indication of competence proven by publications and training courses. The introduction of the Ministry of Health Guidelines regarding the application of Research Projects (DGSAF 0000674-P-16/03/2015), of a declaration of absence of conflict of interest by the scientific Member, arises some interpretation doubts, since any person who belongs to the applicant organization, in theory, could be considered in conflict of interest.

This limitation, substantially, makes it necessary for the AWB to have more than one scientific member, who takes part in the assessment of projects only if not directly involved in the project itself.

Whether within the AWB there must be specific competences on aspects concerning biostatistics or alternate methods is a widely discussed matter. In the first case, more than a necessity for the AWB, it is a responsibility for the Research Project Manager to guarantee the definition of an appropriate and scientifically solid statistics design. The role of the AWB should be more focused on verifying that the statistics design is compatible with the principle of the 3 Rs and, in the event of a doubt, discuss possible improvements with the expert in biostatistics and the Project Manager.

For what concerns the competences on alternative methods, the need arises mainly from the verification that the project Manager has taken into consideration all possible alternatives to using animals, and that there is no other scientifically valid method to achieve the objective of the test. These competences should be present in the curriculum of the above-mentioned members of AWB, as highlighted by the guidelines of the European Commission. A specific competence on the technical aspects of procedures that do not consider the use of animals, can, without any doubt, be interesting, but it must be stressed out that if no animals, no organs, no tissues, or cells of animal origin are used, we leave the field of application of the directive and the responsibilities of the AWB.

The analysis of scientific publications and of the various alternatives in terms of testing models are part of the Project definition process, and the AWB can easily and effectively assess the actual unavoidability of the animal model, by revising the documentation produced by the Project Manager. In any event, the AWB, can collect information independently both from publications and from data bases available at the institutions appointed by the European Commission (European Union Reference Laboratory for alternatives to animal testing—EURL ECVAM) or the local reference centers (In Italy, the Istituto Zooprofilattico Sperimentale della Lombardia e dell'Emilia Romagna—IZSLER).

### Organization of the AWB

One of the aspects still widely controversial is represented by the practical organization of an AWB. Legislative Decree 26/2014 does not regulate on the matter, partly because regulations must be suited in accordance with the type and size of the institutions in which they are to be applied.

As in any other organization, with the aim to continuously improve efficiency and effectiveness, the AWB should set its own Code, transposing the responsibilities provided for by the Directive, in concrete roles within the organization. The Code should define the responsibilities of the various components, the procedures to be followed when recording decisions, the frequency of meetings and the internal revision procedures of research projects, monitoring them after approval by competent authorities and their retrospective assessment. It should define how and how often internal procedures must be monitored, and how to interact with control authorities.

It is necessary to highlight how vital it is to record activities and decisions made, not only for auditing purposes—control authorities must verify the actual activities of the AWB—but also to collect useful data which allow to evaluate possible improvements in the application of the 3 Rs. In addition to recording activities, made up of memoranda and or reports on specific topics, the AWB should define annual goals and make sure said goals are met, with the purpose to monitor the quality of the service. There is no doubt that the AWB is a “service” to the organization that sets it up, and that it should never be considered an obstacle to testing activities or simply a control on the compliance with regulations.

One of the rules that the AWB should set for itself, is the one connected with the turnover of its members, and with the training of new subjects, providing for periods of working under supervision before taking over. The complexity of the roles assigned to the AWB calls for the development of systems that guarantee their operation even in situations of temporary difficulty such as the turnover of personnel or prolonged absences of key figures.

The AWB Code, therefore, constitutes the fundamental element that sets the rules needed to interact with the rest of the organization, including the relations and the communications with control and coordination functions, and the procedures to divulge its activities within the organization (e.g., internal web sites).

## Ethical committees and the AWB

### Ethical committees for animal testing

The opportunity to establish a body in charge of ethical control of activities connected with the use of animals for scientific purposes has been discussed for a long period of time in Italy, since the directive prior to the current one came into force (Legislative Decree 116/1992).

Many institutions introduced internal Ethical Committees for Animal Testing (Comitati Etici per la Sperimentazione Animale, **CESA**), often inspired by the ethical committees for clinical testing or by International Guidelines (Kalman et al., 2011). Not being mandatory by governing authorities, they were voluntary, and had competences of a general nature, mainly focusing on the revision of research projects.

For their own nature (composition, frequency of meetings, professional competence) the CESA represent, in the majority of cases, a valid strategic support but barely able to supply an effective and timely operative support in the practical daily activities of an establishment, as defined by Directive 63/2010/EU and by Legislative Decree 26/2014 and entrusted to the AWB.

The verification of the actual competence of personnel during normal activities, the verification of the correct application of testing procedures on the field, the support to personnel in the definition of the seriousness of test, both prior to and during the test activities (*clinical score*), the implementation of *refinement* techniques or the development of *humane endpoints* call for quick interventions and decisions that only a Body very close to the operative level can guarantee, and cannot be overseen in accordance with the typical approach of the CESAs, which often find themselves acknowledging situations already made obsolete by the quick development of test activities or resolved by decisions already adopted at other levels (e.g., by the Animal Welfare Manager in agreement with the Project Manager).

It is therefore undeniable that the implementation of the AWBs covers some aspects which were not adequately overseen by the CESA, and that the two institutions are completing one another rather than overlapping.

### Interactions between the AWB and ethical committees

Many institutions considered reorganizing the traditional tasks of the CESA assigning them the functions that the law assigns to the AWB, and integrating the AWB members provided for by law inside the CESA. Other institutions, on the other hand, preferred to keep the two bodies separate, assigning CESA a control function over the AWB.

In the latter case, the verification of conformity of research projects to the provisions of Legislative Decree 26/2014, as well as the communication with competent authorities are still assigned to the AWB. CESA control role is implemented by revising the technical-scientific assessment and more generally the admissibility of projects from an ethical point of view.

The critical revision of the actual “justification” on the use of animals, based on the ratio between the expected scientific/educational benefits and the impact on animals' welfare (*harm-benefit ratio*) makes it necessary to be assessed from different points of view, and the contribution of diversified professional figures, such as the ones normally found in ethics committees, becomes an added value.

Having an ethics committee to which to report, on a regular basis, the outcome of monitoring activities of approved projects, the problems encountered, the results achieved, improvement programs, the outcome of interactions with control authorities and the training and development of the application of the 3 Rs. constitutes for the AWB a moment for reflection and exchange of views, and a necessary element for the establishment to be accredited by a Quality Management system. The ethics committee, on its part, can effectively exercise its institutional role, not only by supervising the activities of the AWB, but also by offering support to strategical proposals, such as the ones related to infrastructural interventions or managing the establishments.

There is no doubt that between the AWB and the Ethics Committee there is a fundamental agreement of interests in safeguarding the animals used for testing purposes and at the same time in reaching the best possible results in the research work.

## Open issues and opportunities

### Training of personnel

The assessment of training of personnel and the coherence of their roles concerning the needs of the project, are without a doubt among the open issues in which the AWBs play a fundamental role. There have been many discussions on the training requirements, often just requesting qualifications and attendance certificates to courses and conventions. It is fundamental for the AWB to stress its activity in supporting and monitoring the development of personnel competences on the 3 Rs, using, for lack of local provisions, the indications of the European Commission on the training objectives of personnel and assessment procedures, which once again highlight the fundamental role of AWBs. (National Competent Authorities for the implementation of Directive 2010/63/EU).

### Conflict of interests

The matter concerning potential conflicts of interest for the scientific members belonging to the AWB, but also to the CESA, raises a fundamental question: can the employee of an institution declare not to have generic “conflicts of interest” in assessing the validity of projects (sometimes already financed) of colleagues belonging to the same establishment, with no risk of committing a felony? This declaration (self-certified), not provided for by the Directive, was introduced by Legislative Decree 26/2014 and upon adoption, the definition of “interest” in this specific scope deserves an explanation by competent authorities.

A possible solution, which constitutes the prevailing approach, is that, not being able to assess all possible conflicts of interest, the controller, and the person being controlled must be kept separate and that no direct hierarchical relation can exist between the two.

### Taking on responsibility

An interesting topic is the possibility by the AWBs to make decisions on aspects connected with the management of projects after they have been approved. In many cases, in fact, some issues, not described in the authorization application, but which do not modify either the rigor of the test or the number of animals used, arise during the development of the project. For example, the need to use a different type of animal (e.g., family, gender, or age), or modifications in the test outline (e.g., number of molecules to be tested, number of testing groups, testing *endpoints* observation times, and procedures).

These decisions sometimes even improve the conditions of the animals and it would be advantageous for the AWB to be able to decide to adopt such choices discretionally, duly recording them in its reports and duly notifying control authorities.

Taking on responsibility in these situations would greatly aid not only the effectiveness of safeguarding animals' welfare and research, but it would also increase the AWB credibility toward the institutions they are part of, stressing their pro-active role and not only their role as a first level filter and administrative interface. Because of their function as a link between Project Managers and control Authorities, the AWBs can work effectively only if they are influential and renowned within the institution they belong to.

The taking on of this type of responsibility can be easily verified and assessed by local health authorities, which Legislative Decree 26/2014 already entrust with control function on AWBs work, thus avoiding the adoption of long formal approval procedures, necessary for research projects, even for minor modifications.

### Interaction between the AWBs

The interaction between the AWBs of different institutions, when working on the same project and perhaps needing to perform different procedures on the same animals at different times, is without a doubt an aspect which needs closer examination. It is obvious, that a non-harmonized approach between the different bodies for what concerns the application of the 3 Rs, can only result in negative consequences on the welfare of animals and on the outcome of the research.

The cooperation and sharing of documentation between the various AWBs involved in the Project, is an essential step, but it is also necessary to point out that, in some cases, e.g., international projects, the allocation of responsibilities, especially in “grey areas” such as the transport of animals, is not always clear.

The National Committee for the protection of animals used for scientific purposes could play an important role in coordinating the AWBs, as provided for both by the Directive and by Legislative Decree 26/2014 (art. 38). It is undeniable that a harmonized approach to common issues between different AWBs, can only ease the work of the AWB, of the Project Manager and of Control Authorities, thus avoiding the risk of expressing different opinions or give directions in direct contrast with similar situations.

## Conclusions

The work of the AWBs established in the various establishments is gradually adjusting to the provisions of current laws, but much can be done to optimize the process and guarantee the maximum level of safeguarding animals' welfare, and suiting it to the needs of research at the same time.

A harmonized approach to some critical aspects, such as monitoring procedures, the retrospective assessment of the degree of suffering, the management of research work sometimes in different locations, constitutes an important challenge for every organization operating on test animals, and constitutes—beyond the simple compliance with law requirement—the necessary prerequisite for continuous improvement and the full implementation of the concept behind the 3 Rs. The consolidation of the role of the AWBs and the possibility to effectively interact both inside the establishments and with control authorities and the integration with pre-existing entities, such as the CESA, constitute crucial steps in the implementation of Legislative Decree 26/2014.

## Author contributions

All authors listed have made a substantial, direct and intellectual contribution to the work, and approved it for publication.

### Conflict of interest statement

The authors declare that the research was conducted in the absence of any commercial or financial relationships that could be construed as a potential conflict of interest. The reviewer GP and handling Editor declared their shared affiliation.

## Footnotes

^3^National Competent Authorities for the implementation of Directive 2010/63/EU on the protection of animals used for scientific purposes - A working document on Animal Welfare Bodies and National Committees to fulfil the requirements under the Directive - Brussels, 9-10 October 2014. http://ec.europa.eu/environment/chemicals/lab_animals/pubs_guidance_en.htm.

^4^Legislative Decree 26/2014 - Implementation of Directive 2010/63 / EU on the protection of animals used for scientific purposes; GU n. 61 of March 14, 2014.

## References

[B1] Poirier G. Bergmann C. Denais-Lalieve D. Dontas I. Dudoignon N. Ehall H.. (2015). ESLAV/ECLAM/LAVA/EVERI recommendations for the roles, responsibilities and training of the laboratory animal veterinarian and the designated veterinarian under Directive 2010/63/EU. Lab. Anim. 49, 89–99. 10.1177/0023677214557717 25416607

[B2] Kalman R. Olsson A. S. Bernardi C. van den Broek F. Bronstad A. Gyertyan I. (2011). Ethical evaluation of scientific procedures: recommendations for ethics committees, in The COST Manual of Laboratory Animal Care and Use, eds Howard B. Nevalainen T. Perretta G. (Boca Raton, FL: Taylor and Francis group), 101–129.

